# Resting Metabolic Rate and Substrate Utilization during Energy and Protein Availability in Male and Female Athletes

**DOI:** 10.3390/metabo14030167

**Published:** 2024-03-17

**Authors:** Mahmoud M. A. Abulmeaty, Ali Almajwal, Mervat Elsayed, Heba Hassan, Thamer Alsager, Zaid Aldossari

**Affiliations:** 1 Community Health Sciences Department, College of Applied Medical Sciences, King Saud University, Riyadh 11433, Saudi Arabia; aalmajwal@ksu.edu.sa (A.A.); dr.mervatelsayed@gmail.com (M.E.); dr.hebazaher@gmail.com (H.H.); talsager@ksu.edu.sa (T.A.); zaldossari@ksu.edu.sa (Z.A.); 2 Department of Medical Physiology, School of Medicine, Zagazig University, Zagazig 44519, Egypt

**Keywords:** athletes, energy availability, protein availability, RMR, substrate utilization

## Abstract

Active athletes frequently develop low energy (LEA) and protein availabilities (LPA) with consequent changes in the vital metabolic processes, especially resting metabolic rate (RMR) and substrate utilization. This study investigated the association of energy and protein intakes with RMR and substrate utilization in male and female athletes and those with LEA and LPA. Sixty athletes (35% female, 26.83 ± 7.12 y) were enrolled in this study. Anthropometric measurements and body composition analysis were reported to estimate fat-free mass (eFFM). Dietary intakes were recorded by two-day multiple-pass 24 h recall records and three-day food records and then analyzed by food processor software to calculate protein intake (PI) and energy intake (EI). Indirect calorimetry was used to measure RMR and percentages of substrate utilization. Activity–energy expenditure (AEE) was assessed by using an Actighrphy sensor for three days. Energy availability was calculated using the following formula (EA = EI − AEE/eFFM). The correlation of EI and PI with RMR and substrate utilization was tested with Pearson correlation. In the LEA group, both EI and PI correlated positively with RMR (r = 0.308, 0.355, respectively, *p* < 0.05). In addition, EI showed a positive correlation with the percentage of fat utilization. In the male and sufficient-PA groups, PI correlated positively with the RMR and negatively with the percentage of protein utilization. In conclusion, the percentage of LEA is markedly prevalent in our sample, with a higher prevalence among males. Athletes with LEA had lower fat utilization and lower RMR, while those with sufficient PA showed lower protein utilization with excessive PI. These findings may explain the metabolic responses in the cases of LEA and LPA.

## 1. Introduction

Total energy expenditure (TEE) includes three components in adults, namely basal/resting energy expenditure (BEE), the thermal effect of the food (TEF), and activity–energy expenditure (AEE) [1]. The AEE is further subdivided into non-exercise activity thermogenesis (NEAT) and exercise energy expenditure (EEE) [2]. NEAT is the energy consumed during physical activities other than volitional exercises, such as daily living activities, maintaining posture, and any body movement, making skeletal muscles consume energy above the resting level, usually over 1.6 metabolic equivalents (METs) [3]. Studies investigating AEE in athletes focus on EEE, with an underestimation of the non-exercise physical-activity-related energy expenditure (NEAT).

In humans, TEE is determined by body size and composition, behavior, and environment, i.e., the larger the body—especially with larger fat-free mass (FFM), as is the case for athletes—the higher the energy requirements for maintaining homeostasis, and thus the higher the BEE [2]. In the case of low energy provision, the resting metabolic rate (RMR) is expected to decrease, so the RMR may be considered a proxy for EA [4]. In athletes, considerably high AEE means that the remaining energy available for maintaining homeostasis becomes a critical issue. This is the basis of studying the energy availability (EA) concept in athletes. EA is defined as the remaining energy from energy intake (EI) after subtracting the energy consumed by AEE, adjusted to the FFM of the athlete. The mathematical equation of EA = (EI − AEE)/FFM [5]. The concept of EA is more beneficial than the concept of energy balance when prescribing diets for athletes [6]. Notably, low EA (LEA) can lead to the development of a syndrome called Relative Energy Deficiency in Sports (REDs) [7]. The female athlete triad model with menstrual dysfunction and impaired bone health was the first described form of REDs [8]. In the year 2023, the updated consensus of the International Olympic Committee described REDs as a multifactorial syndrome characterized by impairment of a wide range of physiological and psychological functions in athletes of the male and female sexes that is caused by prolonged and severe exposure to LEA. The disturbed functions include decreased resting metabolic rate (RMR), reproductive function, bone health, cardiovascular health, immune system, and hematological parameters, as well as psychological and behavioral manifestations and negative consequences in performance [9]. The extended model of REDs included the affection of both male and female athletes [7,10]. However, sex differences remain a point for discussion.

Unlike energy provision, athletes usually have a shared belief that they should include sufficient–excessive protein, especially from animal sources, in their diets [11]. According to the American Dietetic Association (ADA) guidelines, protein intake for adult athletes should be 1.2–2.0 g/kg of their body mass. However, many athletes report a higher protein intake than the recommended daily intake (RDI) [12]. The association between high protein intake and protein utilization in the metabolic system of athletes is a questionable point and needs further research.

Indirect calorimetry (IC) is the gold-standard method of RMR measurement and quantifying the percentage of macronutrient utilization. Measuring volumes of respiratory oxygen (VO_2_) and carbon dioxide (VCO_2_) gives the respiratory quotient (RQ), which indicates which substrate is utilized during the test. Consequently, the RQs for carbohydrates, fat, protein, and anaerobes are 1, 0.7, 0.8, and 0, respectively. If a substrate mixture is consumed, then the RQ is 0.8 [13]. Some previous reports have studied the association between low EA and RMR among athletes [14,15]. However, the association of EA and protein availability (PA) with RMR and substrate utilization is lacking, especially in samples that included male and female athletes. Collectively, this study aims to investigate the association of energy and protein provisions with RMR and substrate utilization in male and female athletes and those with LEA and LPA.

## 2. Materials and Methods

### 2.1. Participants and Recruitment

The study sample was recruited from national teams in Saudi Arabia. The scheduled participants (aged 18–45 years) were given an appointment to take study measurements at the nutrition clinic (Nutrition and Metabolism Physiology Clinic) of the College of Applied Medical Sciences, King Saud University. The study was conducted between September 2022 and March 2023. Inclusion criteria included athletes who had allied to a national team or club for at least two years of practicing the sport and underwent regular training for at least 20 h per week [16]. The exclusion criteria included females with amenorrhea or any endocrine disorders. Athletes experiencing eating disorders or psychiatric conditions were also excluded. In addition, athletes with recent injuries or those taking supplements or medications that could affect RMR were excluded. The total sample size of 56 athletes was calculated by G*Power software (version 3.1.9.7; Heinrich-Heine-Universität Düsseldorf, Düsseldorf, Germany) with power (1 − β) = 0.80, significance level (α) = 0.05, and effect size = 0.68 based on the RMR results of Kinoshita et al. [17]. The study protocol was reviewed and approved by the Institutional Review Board (IRB) Committee of the College of Medicine, King Saud University (Reference No. 21/0531/IRB).

### 2.2. Procedures

**Figure 1:**
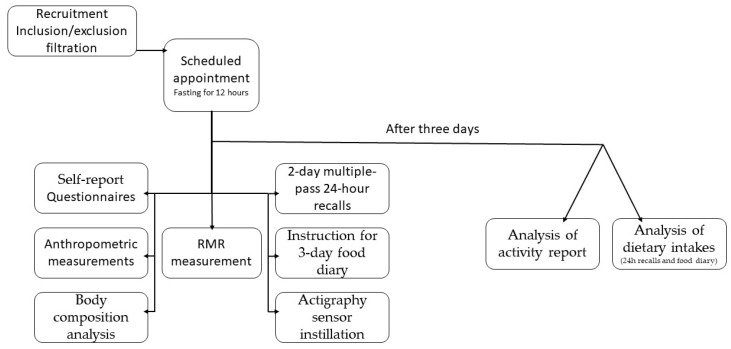
Recruitment and procedures of the current study.

All participants signed an informed consent form after the study was briefly described. Then, participants were subjected to the study protocol, as shown in Figure 1. The study procedures included the measurements outlined in the following subsections.

#### 2.2.1. Self-Report Questionnaires

The questionnaires included demographics, reproductive health history, supplement history, and an eating disorder inventory-2 questionnaire [18].

#### 2.2.2. Anthropometric Measurements

Anthropometric measurements included stature in (cm) and body weight in (kg) by using the Seca scale (SECA Co., Hamburg, Germany). In addition, a non-stretchable tape was used to measure waist and hip circumferences. Body mass index was obtained by dividing weight by stature squared, and the waist–hip ratio was obtained by dividing the waist circumference by the hip circumference. Dominant and non-dominant hand grip strength was measured using the Jamar Hand Grip Dynamometer. An average of three frequent measurements with an outstretched, unsupported arm was used for analysis [19,20].

#### 2.2.3. Body Composition Analysis

Body composition was analyzed using the TANITA BC-418 analyzer (Tanita Co., Tokyo, Japan). The estimated percent body fat (ePBF), fat mass (eFM), eFM index (FM/height squared in meters), estimated fat-free mass (eFFM), eFFM index (FFM/height squared in meters), estimated muscle mass, and total body water (eTBW) were recorded and used for analysis [21].

#### 2.2.4. Dietary Analysis

The dietary data of all athletes were obtained by multiple-pass 24-h recall for two days (one workday and one weekend day preceding the appointment day) in addition to a 3-day food record guided by meal photos (following the appointment day). During the first visit, a research team member interviewed the participants to fill out multiple past 24-h recall forms for two days and then educated the athlete about how to fill out the 3-day food record and send photos of their meals to a WhatsApp number belonging to the research team [22]. Dietary intakes were analyzed by the food processor software (ESHA Research Salem, Salem, OR, USA), which depends on food composition tables from the United States Department of Agriculture. The average of the 5-day analysis (2 days 24 h recall + 3 days food record) was used for statistical analysis. Energy (EI; Kcal/day) and protein (PI; g/day) were reported, and other macronutrient components of the athlete’s diet [23] were used for analysis.

#### 2.2.5. Measuring the RMR

Resting metabolic rate was measured in fasting athletes (for about 12 h) by indirect calorimetry using the Q-NRG+ machine (COSMED, Inc., Albano Laziale, Italy). The testing room was maintained at 25 °C, kept calm, and equipped with a comfortable examination bed.

Resting energy expenditure was measured for about 15 min after excluding the first few minutes. The RMR and percentages of substrate utilization were used for analysis. Division of the CO_2_ produced by the consumed O_2_ results in what is called the respiratory quotient (RQ). The IC calculated fat, CHO, and protein utilization percentages based on RQ [24].

#### 2.2.6. Estimated Activity–Energy Expenditure Monitoring

All participants were instructed to wear a tri-axial accelerometer (ActiGraph wGT3X-BT, Shalimar, FL, USA), which is programmed to record 10 s epochs for three consecutive days, 24 h/day, even during sleep [25]. After finishing the recording period, participants returned to the clinic to analyze the activity pattern and the estimated activity–energy expenditure (AEE) using Actilife software (ActiLife 6.1, ActiGraph, Pensacola, FL, USA). Periods of non-wear time were excluded from the data analysis. The parameters used for statistical analysis included the estimated average activity–energy expenditure (eAEE) (kcal/day), which represents both the non-exercise activity thermogenesis (NEAT) and exercise energy expenditure (EEE). In addition, the software calculates the average eAEE/activity hour (kcal/h), the metabolic equivalent (MET), a count of steps, the steps per minute, and the percentages of total hourly and daily counts per minute (CPM). This was divided into four categories: sedentary time (≤100 CPM), light PA (100–1951 CPM), moderate PA (1952–5724 CPM), and vigorous PA (PA ≥ 5725 CPM) [26].

#### 2.2.7. Energy and Protein Availability

Energy availability was calculated by subtracting the average AEE (kcal/day) from the EI (kcal/day) and dividing the result by the eFFM. Energy availability at less than 30 Kcal/kg eFFM/day was considered low energy availability (LEA) [17]. Protein availability was considered depending on published guidelines of the American Dietetic Association (ADA) [27], i.e., protein availability of less than 1.2 g/kg/day was considered low protein availability (LPA). In addition, protein availability was represented as grams of protein intake per eFFM kg per day (g/kg eFFM/day).

### 2.3. Statistical Analysis

Continuous variables were expressed as means (±SD), while dichotomous variables were expressed as percentages. The normality of variables was tested by the Shapiro–Wilk test. Independent sample t-tests or equivalent non-parametric tests were used to compare the means of the study groups. Cross tabulation with a chi-square test was used to test the difference in categorical variables. Pearson correlation coefficient was used to test the correlation of protein and energy provision with RMR and substrate utilization percentages. All the data were analyzed using SPSS (version 23; SPSS Inc., Chicago, IL, USA).

## 3. Results

### 3.1. Anthropometric and Body Composition Characters of the Development Sample

Sixty athletes were assessed in this study (age 26.83 ± 7.12 years, female = 35%). Table 1 presents the anthropometric and body composition characteristics of the study sample. Male athletes has higher measurements in stature, weight, waist circumference, and hand grips. In addition, eFM, eFMI, and ePBF were higher in female athletes, while eFFM, eFFM, estimated muscle mass, and eTBW were lower in female athletes.

### 3.2. Dietary and Macronutrient Intake in the Study Sample

As shown in Table 2, the average dietary intake and macronutrient composition of the dietary intake were insignificantly different between male and female athletes. The average added sugar was significantly higher in the male group.

### 3.3. Resting Energy Expenditure and Activity–Energy Expenditure Results

The resting energy expenditure indicated by RMR was significantly higher in male athletes, while the percent of protein utilization was higher in the female group. Regarding the activity–energy expenditure, the estimated average AEEs per hour and metabolic equivalents (METs) were significantly higher in male groups. However, the number of step counts was higher in female athletes (Table 3).

### 3.4. Energy and Protein Availability

Female athletes showed significantly higher energy intake per kg of eFFM, higher AEE per kg of eFFM, and higher protein availability per kg of eFFM. In the female group, the energy availability after the exclusion of AEE was 18.85 ± 16.65 Kcal/kg eFFM/day vs. 11.09 ± 13.63 Kcal/kg eFFM/day in the male group, *p* = 0.057 (Table 4). As shown in Table 5, only 32.8% have sufficient energy availability, and 71.4% of the female group has sufficient protein availability. The condition changed significantly in the male group, where only 7.7% had sufficient energy availability, and 66.7% had adequate protein provision. Participants of both the male and female groups practiced similar types of sports.

Data derived from dividing the study sample according to energy and protein availability are shown in Table 6. In the LEA group, protein provision was significantly lower than in the sufficient-EA group. Interestingly, RMR and AEE were significantly higher in the LEA group (*p* < 0.05). Energy availability was significantly low in the low PA group, while RMR and AEE were insignificantly different from the sufficient-PA group.

### 3.5. Correlation of Energy and Protein Availability with RMR and Substrate Utilization

In the LEA group, energy availability correlated positively with RMR and percentage of fat utilization, i.e., the lower the energy intake, the lower the RMR and the lower the fat utilization (Table 7). Furthermore, protein provision correlates positively with the RMR, i.e., in the LEA, the lower the protein intake, the lower the RMR (Table 8). In the group with sufficient protein intake, protein provision correlates positively with RMR and negatively with the percentage of protein utilization, i.e., the higher the protein intake, the higher the RMR, and the lower the protein utilization. Similarly, in the male group, protein provision correlates positively with RMR and negatively with the percentage of protein utilization (Table 8).

## 4. Discussion

This study investigated the association of energy/protein availability with RMR and substrate utilization in male and female athletes. This work answers some tricky questions about energy and protein metabolism in athletes, especially in states of LEA and LPA: Is there a difference between male and female athletes regarding dietary intake, energy availability, protein availability, and substrate utilization? Are athletes more adherent to energy or protein requirements? Is RMR reduced by low energy availability or low protein availability? Which substrate is utilized more in conditions with LEA or LPA?

Regarding sex-related differences in dietary intake in athletes, apart from added sugar, the macronutrient dietary intakes were similar in male and female athletes. This was inconsistent with previous reports, such as Bogdanis et al. [28], who found that male athletes showed a significantly higher average energy intake than female athletes. In another opinion, Nascimento et al. [29] found that both male and female groups reported a low caloric intake with more inadequate protein and saturated fat intake in male athletes after analysis of the dietary intake, as was assessed by 24 h recall. Females have more body fat, so when they become athletes, they suffer from greater social pressure to attain lean and fit bodies, leading them to commit to dietary guidelines more than male athletes. This assumption was supported by our findings, as presented in Table 4 and Table 5. The extensive commitment of female athletes may leave them vulnerable to the development of eating-disorder-like behaviors. De Borja et al. [30] found that the adherence of female athletes to a specific dietary regimen may be associated with behaviors that are consistent with disordered eating. Furthermore, eating disorders (EDs) prevalence is higher in female athletes than in male athletes, ranging from 6 to 45% in female athletes versus 0–19% for male athletes [31]. Notably, our sample was screened for EDs, and all of them were found to be free from EDs.

The current results showed a higher percentage of sufficient protein intake rather than sufficient energy, i.e., 71.4% of the female group had sufficient protein availability versus only 32.8% with sufficient energy availability. In the male group, 66.7% had sufficient protein provision, while only 7.7% had sufficient energy availability. This finding is common in daily practice. Athletes believe that consuming sufficient or excessive protein (double or triple the RDI) rather than eating sufficient carbohydrates and fats is the best practice [12]. During high-intensity physical activity, providing energy, especially energy from carbohydrates and protein, is required to replenish glycogen stores, maintain body weight, and provide adequate protein to repair muscle tissue [32]. The rationale for higher protein intakes than the RDI in athletes is a result of its support for elevated levels of functioning and adaptation to the exercise stimulus [33]. In conditions with mild energy deficits, we can ensure protein requirements increase, which is further increased with excessive exercise [34], i.e., more protein is needed to support gluconeogenesis and increased muscular requirements. It was reported that male athletes consuming 2 g of protein/kg with a non-protein caloric deficit and running 5–10 miles/day still had significant negative nitrogen balance [35].

Interestingly, our results showed that RMR was significantly higher in the LEA group than in the sufficient-EA group. The compensatory mechanisms that provide the required energy for the body systems, especially with increased AEE in the state of LEA, may induce an increase in the RMR. For example, the gluconeogenesis process that produces glucose (the preferred substrate for the metabolism of the nervous tissue) requires more energy; consequently, RMR tends to increase. Kinoshita et al. [17] reported that RMR remains increased in conditions with low EA. Obese non-athletic patients under a very low-calorie ketogenic diet showed an absent reduction in RMR [36]. Another opinion reported a reduction in the RMR during periods with low EA [37]. In addition, Milen et al. [15] found that female athletes with low and reduced EA with or without menstrual disorders had lowered RMR. The causes of discrepancy between the last reports and our results include using the RMR ratio instead of directly measured RMR [37] and using a small sample with only female athletes [15]. The RMR did not change in low- versus sufficient-PA groups.

In the LEA group, the correlation results showed positive correlations between energy intake and RMR, i.e., the lower the energy intake, the lower the RMR, and the lower the fat utilization. Low-fat utilization may conserve the energy needed to utilize fat; consequently, RMR is reduced. This reduction in RMR might be a form of adaptation that acts as an energy-conserving mechanism [38]. This assumption aligns with the updated definition of REDs [9] and with reports from non-athletic adults; RMR showed a significant positive correlation with low carbohydrate scores [39]. In another report, low energy intake could predict future body fat regain, and high energy intake appeared to induce fat loss partly because it was associated with a higher RMR [40]. Furthermore, a lower protein intake was associated with lower RMR.

In the sufficient-PA and male groups, a negative correlation was observed between protein provision and protein utilization, i.e., in athletes with sufficient protein availability, which also had significantly higher energy availability; the higher the protein intake, the lower the protein utilization (oxidized to produce energy). During exercise, skeletal muscles depend on various substrate sources to meet their energy needs. Amino acids represent the lowest percentage of contribution in energy production, i.e., during exercise, protein contribution in ATP production ranges from less than 5% to 15% of TEE in some extreme cases [41]. The factors that affect the percentage of protein utilization during exercise include training status, exercise intensity, and the availability of other substrates (e.g., carbohydrates). From a molecular basis, it was reported that endurance exercise reduces the activation of nuclear factor-κ, leading to mitochondrial adaptations that prevent protein degradation [42]. The substrate utilization in this study depends on the respiratory quotation (RQ) that the IC calculates. Rothschild et al. [43] found that measuring RQ during cycling is affected by many variables, with the most considerable impacts being sex, daily fat and carbohydrate intake, and exercise duration.

## 5. Conclusions

In conclusion, athletes were more committed to protein than energy intake. The percentage of LEA is markedly prevalent in our sample, with more prevalence in males. Athletes with LEA and LPA have distinctive metabolic profiles that should guide us to tailor the best practices. Conditions with LEA showed higher RMR, lower fat utilization, and a positive correlation between energy intake and RMR. In contrast, those with sufficient PA showed lower protein utilization with more protein intake.

## Figures and Tables

**Table 1:** Anthropometric measures and body composition parameters of the study sample.

Variables	Total Sample (*n* = 60) Mean ± SD	Female Athletes (*n* = 21) Mean ± SD	Male Athletes (*n* = 39) Mean ± SD	*p*-Value
Age (years)	26.83 ± 7.12	28.90 ± 7.02	25.71 ± 7.00	0.099
Weight (kg)	70.95 ± 14.64	61.80 ± 9.59	75.87 ± 14.61	**<0.001**
Height (cm)	168.73 ± 7.76	160.81 ± 4.25	173.00 ± 5.53	**<0.001**
Body mass index (kg/m^2^)	24.80 ± 4.14	23.84 ± 3.24	25.32 ± 4.51	0.190
Dominant Average hand grip (kg)	35.80 ± 11.87	23.98 ± 4.94	41.85 ± 9.57	**<0.001**
Non-dominant Average hand grip (kg)	33.93 ± 11.04	22.58 ± 4.80	39.90 ± 8.35	**<0.001**
Waist circumference (cm)	76.95 ± 9.88	72.65 ± 9.39	79.15 ± 9.50	**0.015**
Hip circumference (cm)	100.29 ± 9.81	98.98 ± 11.72	100.96 ± 8.77	0.466
Waist hip ratio	0.77 ± 0.07	0.74 ± 0.06	0.78 ± 0.06	**0.009**
Estimated Percent body fat (%)	20.82 ± 7.84	28.21 ± 6.05	16.84 ± 5.44	**<0.001**
Estimated Fat mass (kg)	14.93 ± 6.61	17.86 ± 6.06	13.34 ± 6.42	**0.010**
Estimated Fat-mass index (kg/m^2^)	5.30 ± 2.42	6.87 ± 2.21	4.46 ± 2.11	**<0.001**
Estimated Fat-free mass (kg)	56.01 ± 11.95	43.89 ± 4.65	62.54 ± 9.24	**<0.001**
Estimated Fat-free mass index (kg/m^2^)	19.49 ± 2.96	16.96 ± 1.53	20.86 ± 2.63	**<0.001**
Estimated Muscle mass (kg)	53.00 ± 11.96	41.67 ± 4.40	59.09 ± 10.15	**<0.001**
Estimated Total body water (kg)	41.01 ± 8.74	32.13 ± 3.40	45.79 ± 6.74	**<0.001**

Bold *p*-values indicate statistical significance.

**Table 2:** Dietary intake and its macronutrient composition of the study sample.

Variables	Total Sample (*n* = 60) Mean ± SD	Female Athletes (*n* = 21) Mean ± SD	Male Athletes (*n* = 39) Mean ± SD	*p*-Value
Average daily energy intake (Kcal/day)	1911.00 ± 690.86	1919.45 ± 694.34	1906.43 ± 698.03	0.099
Average daily protein intake (g/day)	99.90 ± 39.11	91.66 ± 33.44	104.34 ± 41.58	0.234
Average daily CHO intake (g/day)	222.44 ± 96.77	211.94 ± 95.55	228.10 ± 98.18	0.542
Average daily fiber intake (g/day)	13.87 ± 7.52	16.01 ± 9.68	12.72 ± 5.88	0.106
Average daily total sugar intake (g/day)	77.82 ± 49.87	67.61 ± 39.29	83.31 ± 54.41	0.248
Average daily added sugars (g/day)	27.75 ± 34.15	15.30 ± 17.92	34.86 ± 38.86	**0.037**
Average daily fat intake (g/day)	71.69 ± 37.27	81.60 ± 39.43	66.35 ± 35.43	0.132
Average daily sat. Fat intake (g/day)	25.00 ± 16.99	28.25 ± 18.66	23.24 ± 15.98	0.280
Average daily MUF intake (g/day)	14.56 ± 9.59	15.38 ± 12.46	14.13 ± 7.88	0.641
Average daily PUF intake (g/day)	8.10 ± 5.27	9.05 ± 6.72	7.61 ± 4.37	0.326
Average daily trans fat intake (g/day)	0.43 ± 0.60	0.23 ± 0.31	0.54 ± 0.69	0.061

CHO = carbohydrates; MUF = monounsaturated fatty acids; PUF = polyunsaturated fatty acids; bold *p*-values indicate statistical significance.

**Table 3:** Indirect calorimetry and actigraphy parameters of the study participants.

Variables	Total Sample (*n* = 60) Mean ± SD	Female Athletes (*n* = 21) Mean ± SD	Male Athletes (*n* = 39) Mean ± SD	*p*-Value
Measured RMR (kcal/day)	1786.73 ± 362.02	1503.19 ± 188.49	1939.41 ± 341.22	**<0.001**
Percent of utilized fat (%)	55.28 ± 23.00	51.70 ± 24.13	57.21 ± 22.46	0.382
Percent of utilized carbohydrates (%)	25.22 ± 23.65	25.57 ± 25.50	25.04 ± 22.94	0.935
Percent of utilized protein (%)	19.65 ± 4.13	23.16 ± 3.77	17.76 ± 2.92	**<0.001**
Estimated average AEE/day (kcal/day)	1288.41 ± 532.77	1153.93 ± 421.69	1360.83 ± 575.96	0.153
Estimated average AEE/hour (kcal/h)	73.90 ± 27.18	62.79 ± 22.75	79.87 ± 27.74	**0.019**
Metabolic equivalent (METs)	1.45 ± 0.17	1.38 ± 0.17	1.48 ± 0.17	**0.030**
% of time in light activity (%)	77.62 ± 6.52	77.17 ± 8.38	77.87 ± 5.38	0.695
% of time in moderate activity (%)	22.27 ± 6.79	22.59 ± 8.86	22.10 ± 5.49	0.791
% of time in vigorous activity (%)	0.07 ± 0.33	0.14 ± 0.47	0.04 ± 0.22	0.244
% of time in very vigorous activity (%)	0.04 ± 0.21	0.10 ± 0.35	0.00 ± 0.00	0.068
Count of steps (step/day)	42,800.13 ± 24,064.55	55,2796.8 ± 30,062.1	37,417.3 ± 18,399.0	**0.017**
Steps per min (step/min)	10.06 ± 3.51	10.34 ± 4.28	9.90 ± 3.06	0.650

RMR = resting metabolic rate; AEE = activity–energy expenditure; bold *p*-values indicate statistical significance.

**Table 4:** Energy and protein availability parameters.

Variables	Total Sample (*n* = 60) Mean ± SD	Female Athletes (*n* = 21) Mean ± SD	Male Athletes (*n* = 39) Mean ± SD	*p*-Value
Energy intake (Kcal/kg eFFM/day)	35.43 ± 14.47	43.79 ± 14.94	30.92 ± 12.17	0.001
Estimated AEE (Kcal/kg eFFM/day)	23.16 ± 8.30	26.11 ± 8.64	21.57 ± 7.77	0.043
Energy availability (Kcal/kg eFFM/day)	13.81 ± 15.09	18.85 ± 16.65	11.09 ± 13.63	0.057
Protein availability (g/kg/day)	1.40 ± 0.44	1.46 ± 0.40	1.37 ± 0.46	0.446
Protein availability (g/kg eFFM/day)	1.79 ± 0.62	2.06 ± 0.65	1.65 ± 0.56	0.012

eFFM = estimated fat-free mass; AEE = activity–energy expenditure.

**Table 5:** The study participants’ sports types and energy and protein availability categories.

Variables	Female Athletes (*n* = 21) % within Variable (% within the Group)	Male Athletes (*n* = 39) % within Variable (% within the Group)	*p*-Value
Energy availability			0.080
Sufficient energy availability	62.5 (32.8)	37.5 (7.7)	
Low energy availability	30.8 (76.2)	69.2 (92.3)	
Protein availability			0.705
Sufficient protein availability	36.6 (71.4)	63.4 (66.7)	
Low protein availability	31.6 (28.6)	68.4 (33.3)	
Sports			0.127
Bodybuilding	22.2 (9.5)	77.8 (17.9)	
Powerlifting	33.3 (9.5)	66.7 (10.3)	
Spinning	100.0 (4.8)	0.0 (0.0)	
Basketball	100 (2.5)	0 (0)	
CrossFit	0.0 (0.0)	100.0 (12.8)	
Martial arts	100 (4.8)	0.0 (0.0)	
Tennis	100 (4.8)	0.0 (0.0)	
Football	39.1 (42.9)	60.9 (35.9)	
Weight-lifting	100.0 (9.5)	0.0 (0.0)	
Beach volleyball	0 (0)	100.0 (2.6)	
Karate	0 (0)	100.0 (2.6)	
Cycling	22.2 (9.5)	77.8 (17.9)	
Judo	100 (4.8)	0.0 (0.0)	
Sport type			0.740
Individual sport	33.3 (57.1)	66.7 (61.5)	
Team sport	37.5 (42.9)	62.5 (38.5)	

**Table 6:** Characteristics of study sample grouping according to energy and protein availability.

Variables	Energy Availability		*p*-Value	Protein Availability		*p*-Value
	Sufficient EA (*n* = 8) Mean ± SD	Low EA (*n* = 52) Mean ± SD		Sufficient PA (*n* = 41) Mean ± SD	Low PA (*n* = 19) Mean ± SD	
Age (year)	25.00 ± 9.00	27.12 ± 6.85	0.439	27.56 ± 7.52	25.26 ± 6.05	0.248
Gender (female %)	62.50	30.80	0.080	36.6	31.6	0.705
EA (Kcal/kg eFFM/day)	45.53 ± 12.52	8.93 ± 7.72	<0.001	17.48 ± 16.14	5.89 ± 8.39	0.005
PA (g/kg/day)	1.78 ± 0.34	1.34 ± 0.43	0.008	1.62 ± 0.32	0.91 ± 0.20	<0.001
RMR (Kcal/day)	1538.38 ± 210.86	1824.94 ± 366.54	0.036	1800.24 ± 397.84	1757.58 ± 276.68	0.675
Fat utilization (%)	43.55 ± 25.26	57.08 ± 22.35	0.122	58.52 ± 21.40	48.29 ± 25.32	0.110
CHO utilization (%)	34.29 ± 26.91	23.83 ± 23.08	0.248	21.97 ± 21.65	32.26 ± 26.74	0.132
Protein utilization (%)	22.16 ± 3.63	19.26 ± 4.09	0.064	19.74 ± 4.67	19.45 ± 2.70	0.118
AEE (Kcal/day)	886.62 ± 336.66	1350.23 ± 532.53	0.021	1323.03 ± 574.44	1213.71 ± 434.11	0.805

EA = energy availability; PA = protein availability; RMR = resting metabolic rate; CHO = carbohydrate; AEE = activity–energy expenditure.

**Table 7:** Correlation of RMR and substrate utilization with energy availability in the study sample grouping according to energy and protein availability.

Variables		Energy Availability		Protein Availability		Gender	
		Sufficient EA (*n* = 8)	Low EA (*n* = 52)	Sufficient PA (*n* = 41)	Low PA (*n* = 19)	Females (*n* = 21)	Males (*n* = 39)
RMR (Kcal/day)	r	0.501	0.308 *	0.032	−0.194	−0.058	0.084
	*p*-Value	0.206	0.027	0.844	0.426	0.801	0.612
Fat utilization (%)	r	0.135	0.312 *	−0.158	0.279	0.178	0.001
	*p*-Value	0.749	0.024	0.323	0.248	0.441	0.998
CHO utilization (%)	r	−0.066	−0.246	0.179	−0.281	−0.172	0.020
	*p*-Value	0.876	0.079	0.262	0.244	0.457	0.902
Protein utilization (%)	r	−0.450	−0.263	−0.096	0.170	0.095	−0.159
	*p*-Value	0.289	0.059	0.551	0.488	0.684	0.335

EA = energy availability; PA = protein availability; CHO = carbohydrates; bold *p*-values indicate statistical significance; * correlation is significant at the 0.05 level (2-tailed).

**Table 8:** Correlation of RMR and substrate utilization with protein availability in the study sample grouping according to energy and protein availability.

Variables		Energy Availability		Protein Availability		Gender	
		Sufficient EA (*n* = 8)	Low EA (*n* = 52)	Sufficient PA (*n* = 41)	Low PA (*n* = 19)	Females (*n* = 21)	Males (*n* = 39)
RMR (Kcal/day)	r	0.477	0.355 *	0.451 **	−0.094	0.105	0.319 *
	*p*-Value	0.232	0.010	0.003	0.701	0.650	0.048
Fat utilization (%)	r	0.121	0.253	0.111	0.020	0.048	0.259
	*p*-Value	0.776	0.071	0.490	0.934	0.835	0.111
CHO utilization (%)	r	−0.050	−0.188	−0.009	−0.027	−0.032	−0.208
	*p*-Value	0.907	0.182	0.954	0.911	0.889	0.204
Protein utilization (%)	r	−0.473	−0.263	−0.420 **	0.080	0.115	−0.358 *
	*p*-Value	0.263	0.060	0.006	0.746	0.621	0.025

EA = energy availability; PA = protein availability; CHO = carbohydrates; bold *p*-values indicate statistical significance; * correlation is significant at the 0.05 level (2-tailed); ** correlation is significant at the 0.01 level (2-tailed).

## Data Availability

Original data supporting these results are available on request from the corresponding author for reasonable purposes based on the regulations of the Leaders Development Institute under the Ministry of Sport in Saudi Arabia.
